# Vitamin D Binding Protein and Monocyte Response to 25-Hydroxyvitamin D and 1,25-Dihydroxyvitamin D: Analysis by Mathematical Modeling

**DOI:** 10.1371/journal.pone.0030773

**Published:** 2012-01-24

**Authors:** Rene F. Chun, Bradford E. Peercy, John S. Adams, Martin Hewison

**Affiliations:** 1 UCLA and Orthopaedic Hospital, Department of Orthopaedic Surgery and Orthopaedic Hospital Research Center, Los Angeles, California, United States of America; 2 Department of Mathematics and Statistics, University of Maryland, Baltimore County, Baltimore, Maryland, United States of America; 3 Molecular Biology Institute, University of California Los Angeles, Los Angeles, California, United States of America; Roswell Park Cancer Institute, United States of America

## Abstract

Vitamin D binding protein (DBP) plays a key role in the bioavailability of active 1,25-dihydroxyvitamin D (1,25(OH)_2_D) and its precursor 25-hydroxyvitamin D (25OHD), but accurate analysis of DBP-bound and free 25OHD and 1,25(OH)_2_D is difficult. To address this, two new mathematical models were developed to estimate: 1) serum levels of free 25OHD/1,25(OH)_2_D based on DBP concentration and genotype; 2) the impact of DBP on the biological activity of 25OHD/1,25(OH)_2_D *in vivo*. The initial extracellular steady state (eSS) model predicted that 50 nM 25OHD and 100 pM 1,25(OH)_2_D), <0.1% 25OHD and <1.5% 1,25(OH)_2_D are ‘free’ *in vivo*. However, for any given concentration of total 25OHD, levels of free 25OHD are higher for low affinity versus high affinity forms of DBP. The eSS model was then combined with an intracellular (iSS) model that incorporated conversion of 25OHD to 1,25(OH)_2_D via the enzyme CYP27B1, as well as binding of 1,25(OH)_2_D to the vitamin D receptor (VDR). The iSS model was optimized to 25OHD/1,25(OH)_2_D-mediated *in vitro* dose-responsive induction of the vitamin D target gene cathelicidin (CAMP) in human monocytes. The iSS model was then used to predict vitamin D activity *in vivo* (100% serum). The predicted induction of CAMP *in vivo* was minimal at basal settings but increased with enhanced expression of VDR (5-fold) and CYP27B1 (10-fold). Consistent with the eSS model, the iSS model predicted stronger responses to 25OHD for low affinity forms of DBP. Finally, the iSS model was used to compare the efficiency of endogenously synthesized versus exogenously added 1,25(OH)_2_D. Data strongly support the endogenous model as the most viable mode for CAMP induction by vitamin D *in vivo*. These novel mathematical models underline the importance of DBP as a determinant of vitamin D ‘status’ *in vivo*, with future implications for clinical studies of vitamin D status and supplementation.

## Introduction

In recent years there has been a surge of interest in vitamin D and its wide-ranging health benefits. This is due, in part, to the many association studies linking vitamin D status with common human diseases [1], [2]. However, another key factor that has influence our current view of vitamin D and human health has been the reappraisal of vitamin D physiology that has taken place over the last five years [2]. Two pivotal concepts are central to our new perspective on vitamin D: 1) it is now clear that sub-optimal vitamin D status or vitamin D insufficiency is a prevalent health problem across the globe [1]; and 2) vitamin D is a potent modulator of biological responses that extend far beyond its traditional effects on calcium homeostasis and bone metabolism [3], [4], [5], [6].

This new perspective on vitamin D is highly dependent on analysis of serum concentrations of 25-hydroxyvitamin D (25OHD), which is considered to be the most dependable marker of vitamin D status in any given individual [7]. Serum 25OHD levels have been used widely in disease association studies but the precise values that define vitamin D-sufficiency and insufficiency remain controversial [8]. In this study we have investigated another component of the vitamin D system that further complicates the analysis of vitamin D status, namely the serum vitamin D binding protein (DBP). DBP is the main serum carrier of vitamin D metabolites with albumin acting as an alternative lower affinity binder [9], [10]. DBP exists in three major polymorphic forms, yielding six allelic combinations that occur at different frequencies among ethnic groups [11]. The different allelic forms of DBP circulate at varying concentrations [12], and exhibit different binding affinities for 25OHD and 1,25(OH)_2_D [13]. Both of these variables have the potential to influence the bioavailability of vitamin D, with recent studies suggesting that some functions of vitamin D correlate more closely with levels of ‘free’ 25OHD rather than the total serum concentrations of this metabolite [14].

In addition to its transport function, DBP also plays a key role in the endocrine synthesis of 1,25(OH)_2_D within renal proximal tubules, where 25OHD bound to DBP is actively recovered from glomerular filtrate via megalin-mediated receptor endocytosis [15]. This mechanism fuels the metabolism of 25OHD by kidney cells expressing the vitamin D-activating enzyme 25-hydroxyvitamin D-1α-hydroxylase (CYP27B1) but also acts to maintain serum levels of 25OHD. In contrast to its actions in the kidney, the role of DBP as a mediator of vitamin D metabolism and function in other target cells remains far less clear, despite the fact that CYP27B1 expression has been described for a wide range of extra-renal tissues [16]. Data from our group have shown that local intracrine conversion of 25OHD to 1,25(OH)_2_D in monocytes expressing CYP27B1 and the vitamin D receptor (VDR) is a key mechanism underpinning innate antibacterial responses [17]. The efficacy of this activity is highly dependent on the availability of substrate for CYP27B1, namely serum levels of 25OHD [17], [18]. However, studies *in vitro* have shown that 25OHD-induced monocyte-macrophage antibacterial activity is attenuated by the presence of DBP, with this effect being most pronounced with high affinity forms of DBP [19]. Similar effects have also been demonstrated in keratinocytes for 1,25(OH)_2_D induced responses [20]. The conclusion from these studies is that non-classical target cells for vitamin D such as monocytes-macrophages are dependent on ‘free’, rather than DBP-bound vitamin D ligands. This supports the so-called ‘free hormone hypothesis’ for the action of steroid hormones in general [21] but also suggests that the definition of vitamin D status cannot simply be defined by total serum levels of 25OHD.

In the current study, we have explored further the importance of DBP as a determinant of free vitamin D and vitamin D function. Given that physical analysis of free levels of 25OHD or 1,25(OH)_2_D in serum is extremely difficult, we have used a mathematical extra-cellular steady state (eSS) model to estimate free levels of these metabolites based on concentration and genotype-defined variations in DBP affinity. The eSS model was then extended to assess the impact of DBP on intracellular responses to 25OHD and 1,25(OH)_2_D, using an intracellular steady state (iSS) model validated by *in vitro* dose-response studies with adherent monocytes-macrophages. Using this approach, we projected the effects of DBP genotype/affinity on non-classical responses to vitamin D *in vivo*. This DBP mathematical model provides an important new tool for further analysis of the cellular actions of vitamin D but may also help to redefine parameters for vitamin D status used in clinical studies.

## Results

### A mathematical model for estimation of ‘free’ 25OHD and 1,25(OH)_2_D based on DBP concentration and affinity

**Figure 1:**
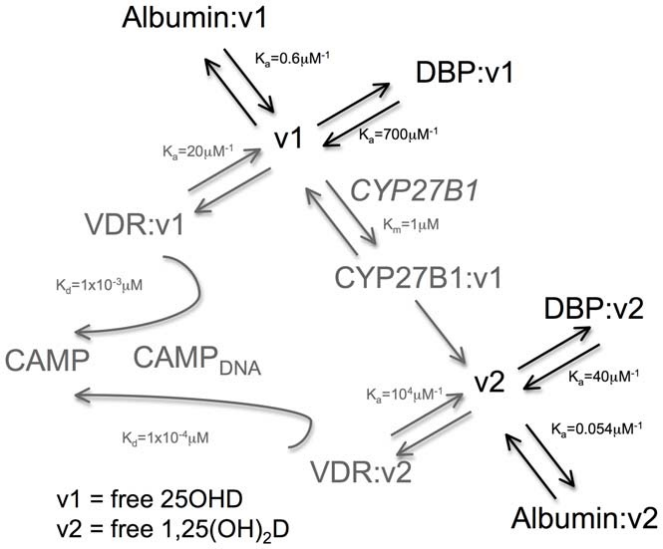
Schematic framework of parameters used to produce extracellular steady state (eSS) and intracellular (iSS) mathematical models for vitamin D metabolism and function. Free 25OHD and 1,25(OH)_2_D interacting with extra-cellular vitamin D binding protein (DBP) or albumin indicated in black text and arrows (eSS model). Intra-cellular interactions involving the vitamin D-activating enzyme (CYP27B1), the vitamin D receptor (VDR) and transcriptional induction of the antibacterial protein CAMP via interaction between VDR and the CAMP gene promoter (CAMP-DNA) indicated by grey text and arrows (iSS model).

**Figure 2:**
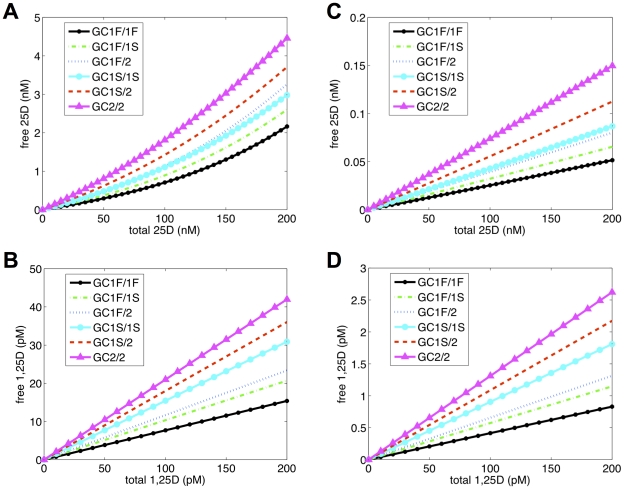
Effects of DBP genotype on free 25OHD and 1,25(OH)_2_D *in vitro* and *in vivo*. eSS-predicted levels of free 25OHD and 1,25(OH)_2_D relative to total serum levels of these metabolites for in vitro tissue culture conditions (5% serum) and in vivo (100% serum) according to DBP genotype (GC allele combinations). X-axis indicates total serum concentrations of 25OHD (nM) or 1,25(OH)_2_D (pM) and Y-axis indicates concentration of free 25OHD or 1,25(OH)_2_D. Concentration of (A) 1,25(OH)_2_D = 5 pM (5% serum), (B) 25OHD = 2.5 nM (5% serum), (C) 1,25(OH)_2_D = 100 pM (100% serum) or (D) 25OHD = 50 nM (100% serum) were fixed.

Previous studies have described mathematical models to estimate serum levels of ‘free’ (unbound) 25OHD and 1,25(OH)_2_D, based on two-ligand-two-binding protein ‘steady-state’ parameters [10], [22], [23] (Figure 1 dark text and arrows). In each case, these models utilized a single binding coefficient for DBP binding of 25OHD and 1,25(OH)_2_D. However, this does not reflect the natural variations in DBP binding affinity that occur as a consequence of group-specific component (GC) allelic inheritance (Table 1). Therefore, we generated a new model for determining free vitamin D metabolites that incorporated DBP affinity coefficients for the six different GC allele combinations described in Table 1. The resulting eSS model was used to predict levels of free 25OHD and 1,25(OH)_2_D *in vitro* (5% serum) (Figure 2A and 2B) and *in vivo* (100% serum) (Figure 2C and 2D). In each case, an appropriate concentration of serum DBP and albumin was assigned to each *in vitro* or *in vivo* condition and genotype combination. Likewise, levels of 25OHD (2.5 nM *in vitro* and 50 nM *in vivo*) and 1,25(OH)_2_D (5 pM *in vitro* and 100 pM *in vivo*) were fixed when concentrations of the other metabolite varied. Data showed that for a physiological level of serum 25OHD (50 nM), the percentage of free 25OHD varied between 0.5–1.5% *in vitro*, and 0.026–0.074% *in vivo* depending on DBP genotype. For a physiological level of 1,25(OH)_2_D (100 pM), the level of free 1,25(OH)_2_D varied between 7.5–22% *in vitro*, and 0.4–1.3% *in vivo*. In each case, the highest level of free 25OHD or 1,25(OH)_2_D was observed for the GC allelic combination with the lowest binding affinity, GC2/2. To further illustrate the impact of DBP genotype on levels of free 25OHD *in vivo*, data were generated for each combination of GC alleles under conditions of vitamin D-deficiency (25 nM total 25OHD), -sufficiency (50 nM 25OHD) and enhanced-sufficiency (100 nM 25OHD). Results in Table 2 showed a sustained 3-fold difference in free levels of 25OHD for low affinity GC2-2 DBP versus GC1F-1F DBP across the spectrum of vitamin D status.

**Table 1:** Binding protein and ligand biochemical parameters for eSS mathematical model.

Average human serum levels [23]		Association constants (Ka) [9], [10]	
DBP (mixed)	5.0 µM	DBP for 25OHD	7×10^8^ M^−1^
Albumin	650 µM	DBP for 1,25(OH)_2_D	4×10^7^ M^−1^
25OHD	50 nM	Albumin for 25OHD	6×10^5^ M^−1^
1,25(OH)_2_D	0.1 nM	Albumin for 1,25(OH)_2_D	5.4×10^4^ M^−1^

DBP concentration				
by genotype (average) [12]		Relative affinity [13]	25OHD	1,25(OH)_2_D
GC1F/1F	5.17 µM	GC1F	1.000	1.000
GC1F/1S	5.15 µM	GC1S	0.536	0.356
GC1S/1S	5.27 µM	GC2	0.321	0.233
GC1F/2	4.77 µM			
GC1S/2	4.79 µM			
GC2/2	4.35 µM			

**Table 2:** Predicted impact of vitamin D status and DBP genotype (Gc allelic combinations) on free 25OHD as projected by eSS model.

	Vitamin D		Vitamin D		Vitamin D	
	Deficiency		Sufficiency		Sufficiency (higher)	
Subject	Total	Free	Total	Free	Total	Free
Genotype	25OHD (nM)	25OHD (nM)	25OHD (nM)	25OHD (nM)	25OHD (nM)	25OHD (nM)
GC1F/1F	25	0.006	50	0.013	100	0.025
GC1F/1S	25	0.008	50	0.016	100	0.032
GC1F/2	25	0.010	50	0.019	100	0.039
GC1S/1S	25	0.011	50	0.021	100	0.043
GC1S/2	25	0.014	50	0.028	100	0.055
GC2/2	25	0.018	50	0.037	100	0.074

### A mathematical model for estimation of intracellular responses to 25OHD and 1,25(OH)_2_D

The potential importance of free 25OHD as a measurement of the bioavailability and function of this metabolite has been highlighted by recent studies of skeletal homeostasis [14], a classical function of vitamin D. Additionally, data from our group indicate that free 25OHD may also be the key determinant of non-classical responses to vitamin D [19]. Therefore, to assess the biological impact of free 25OHD and 1,25(OH)_2_D, we extended the mathematical modeling derived from the initial DBP/albumin binding coefficients (eSS model) to include parameters for 25OHD metabolism and 1,25(OH)_2_D receptor binding (Figure 1 gray text and arrows). The resulting intracellular iSS model incorporated the following considerations: 1) movement of vitamin D metabolites from the extracellular space into intracellular fluid; 2) enzymatic conversion of 25OHD into 1,25(OH)_2_D via the enzyme CYP27B1; 3) binding of 25OHD and 1,25(OH)_2_D to vitamin D receptor (VDR); and 4) liganded-VDR binding to vitamin D response elements in target gene promoters, leading to active transcription. Table 3 and 4 provide a brief description of the variables and parameters used in the models. The mathematical equations used to express the relationships outlined in the full Figure 1 (eSS and iSS models) are described in the Mathematical Modeling subsection of Material and Methods along with more detailed justifications for parameters.

**Table 3:** Variables for mathematical modeling.

Variable	Symbol	Variable	Symbol
Free 25OHD (intracellular)	v_1_^c^	VDR:25OHD complex	r_1_
Free 25OHD (extracellular)	v_1_^o^	VDR:1,25(OH)_2_D complex	r_2_
Free 1,25(OH)_2_D (intracellular)	v_2_^c^	CYP27B1:25OHD	Y_1_
Free 1,25(OH)_2_D (extracellular)	v_2_^o^	Transactivation signal	CAMP
Total VDR	R_T_	VDRE activated by r_1_	o_1_
Total CYP27B1	Y_T_	VDRE activated by r_2_	o_2_

**Table 4:** Parameters for iSS mathematical model.

Value	(Unit) Function	Rationale
d = 6	(hr^−1^) permeability of cells to free 25OHD or 1,25(OH)_2_D	fit from i
K_r1_ = 5×10^−2^	(µM) rate constant v_1_^c^ binding to VDR	ii
K_r2_ = 1×10^−4^	(µM) rate constant v_2_^c^ binding to VDR	iii
K_cat_ = 1×10^−3^	(hr^−1^) activating constant for 25OHD:CYP27B1	iv
K_m_ = 1	(µM) Michaelis constant for 25OHD binding to CYP27B1	v
Y_T_ = 3.0×10^−4^	(µM) total concentration of CYP27B1	estimate & iv
η = 1	(µM) net CAMP production	normalized
R_T_ = 1.2×10^−3^	(µM) concentration of VDR	vi
K_cc1_ = 1×10^−3^	(µM) VDR:25OHD affinity for CAMP VDRE	vii
K_cc2_ = 1×10^−4^	(µM) VDR:1,25(OH)_2_D affinity for CAMP VDRE	viii & [44]
mm = 1	(none) cooperativity constant for 25(OH)_2_D binding by VDR	viii
pp = 2	(none) cooperativity constant for 1,25(OH)_2_D binding by VDR	viii
m = 2	(none) cooperativity constant for VDRE binding by r_1_	viii
p = 2	(none) cooperativity constant for VDRE binding by r_2_	viii

i. rate has only been measured for 1,25(OH)_2_D [50].

ii. K_r1_ = 500*K_r2_.

iii. K_r2_ = 1/K_d_ where K_d_ = 1×10^−10^ [42].

iv. K_cat_*Y_T_ = 0.1 µM/hr [46].

v. estimate based on [29], [47], [48], [49].

vi. 3000 molecules/cell [40] and spherical cell of 10 µm radius [41].

vii. K_cc1_ = 10*K_cc2_.

viii. fit to in vitro data.

**Figure 3:**
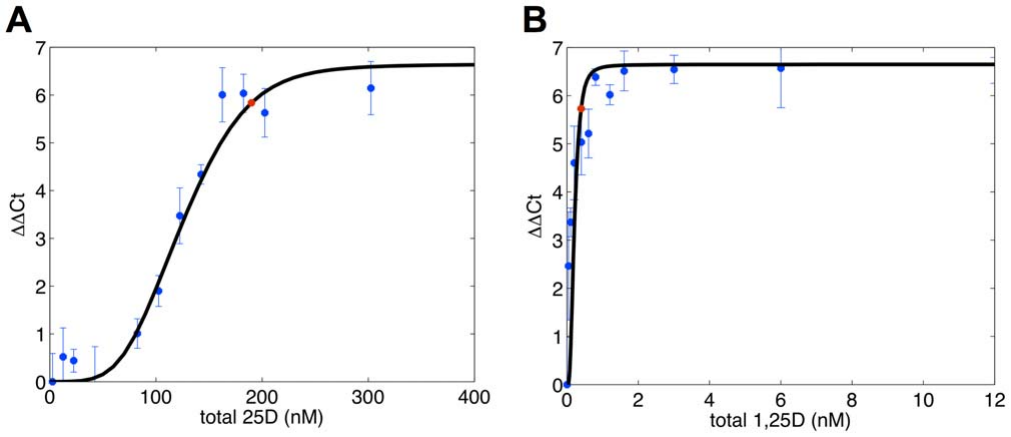
Comparison of iSS-predicted effects of 25OHD or 1,25(OH)_2_D on monocyte expression of CAMP with observed *in vitro* responses of monocytes to treatment with these metabolites. Adherent human monocytes were incubated for 6 hrs in media containing 5% serum with doses of (A) 25OHD (1–300 nM) and (B) 1,25(OH)_2_D (0.1–6 nM). The experimental data is indicated by blue dots and error bars (± SD). Black lines indicate data predicted by the iSS mathematical model assuming basal levels of VDR and CYP27B1 (i.e. no activation). For the purpose of this modeling, DBP was represented by the GC1F/1F allelic combination.

The development of the iSS model was based on data from *in vitro* (5% human serum) analysis of the dose-responsive effects of 25OHD and 1,25(OH)_2_D on monocyte expression of mRNA for the antibacterial protein cathelicidin (CAMP). Initial analysis using the iSS model was based on a single DBP genotype (GC1F/1F) at a fixed concentration of 0.25 µM (5% serum). The resultant modeling is shown in Figure 3. The experimental data from the *in vitro* dose-response study is represented by the blue dots while the black lines represent the values predicted by the iSS model. Based on these observations, the iSS model was then used to predict the induction of monocyte-macrophage CAMP by 25OHD *in vivo* relative to vitamin D status (deficiency [25 nM 25OHD], sufficiency [50 nM 25OHD], and higher sufficiency [100 nM 25OHD]) and DBP genotype (using corresponding affinity constants [Table 1]) and raising the concentrations of DBP and albumin from 5% serum to 100% serum conditions. The resulting data (Table 5), indicate that under the same basal conditions used for *in vitro* data in Figure 3, the iSS model predicts only a minimal induction of *in vivo* CAMP expression, with this being unaffected by DBP genotype. We have shown previously that vitamin D-mediated induction of monocyte CAMP is potently enhanced following the induction of CYP27B1 and VDR by pathogen-associated molecular patterns (PAMPs) such as 19 kDa lipoprotein (toll-like receptor [TLR]2 ligand) or lipopolysaccharide (TLR4 ligand) [17], [18]. Therefore, additional iSS data were generated incorporating a 5-fold induction of VDR and a 10-fold induction of CYP27B1 expression, similar to those described in other studies [24], [25]. Under these conditions of VDR/CYP27B1 activation, the iSS model predicted a 3- to 7-fold induction of CAMP at 50 nM 25OHD for low affinity forms of DBP (GC1S/2 or GC2/2), with this increasing to 20–40-fold at 100 nM 25OHD. By contrast, for high affinity forms of DBP (GC1F/1F) the predicted induction of CAMP by 25OHD remained minimal even at levels of 25OHD defined as vitamin D-sufficient (50 nM) (Table 5). For this particular DBP genotype, a meaningful rise in CAMP induction was only observed at 100 nM serum 25OHD.

**Table 5:** Predicted effects of vitamin D status and DBP genotype (Gc allelic combinations) on *in vivo* monocyte expression of CAMP under basal or immune activated conditions.

	Deficiency		Sufficiency		Sufficiency (higher)	
Subject	25 nM 25OHD (total)		50 nM 25OHD (total)		100 nM 25OHD (total)	
Genotype	Basal	Activated	Basal	Activated	Basal	Activated
GC1F/1F	0.010	0.010	0.010	0.011	0.010	0.019
GC1F/1S	0.010	0.010	0.010	0.012	0.010	0.035
GC1F/2	0.010	0.011	0.010	0.015	0.010	0.062
GC1S/1S	0.010	0.011	0.010	0.019	0.010	0.090
GC1S/2	0.010	0.013	0.010	0.032	0.010	0.199
GC2/2	0.010	0.018	0.010	0.072	0.010	0.420

The adaptability of the mathematical model was tested relative to *in vitro* induction of the osteocalcin gene in MG-63 human osteoblastic bone cells (see file Text S1). After incubation for 6 hrs in media containing 2% serum with doses of 25OHD (0–200 nM) or 1,25(OH)_2_D (0–20 nM), RNA was isolated, cDNA synthesized and osteocalcin expression (ΔΔCt) determined by qPCR (Figure S1). The dashed lines indicate data produced by the iSS mathematical model using monocyte parameters. Given the different cellular context and kinetics of activating a different gene, not surprisingly, the data generated by the monocyte model parameters (dashed lines) did not match the experimental data for osteoblastic cells (blue dots with error bars). Since 1,25(OH)_2_D activation is mechanistically the most direct pathway of action, parameters pertaining to 1,25(OH)_2_D interactions were modified first. The less pronounced rise in osteocalcin expression in response to escalating doses of 1,25(OH)_2_D strongly indicated that MG63 cells were markedly less sensitive to 1,25(OH)_2_D compared to adherent monocytes. Mathematically, this was most effectively expressed with a reduction in *K_r2_*, the affinity of VDR for 1,25(OH)_2_D parameter. Adjustments in the *K_cc2_* (1,25(OH)_2_D/VDR affinity for VDRE-DNA parameter), *pp* (cooperativity constant of the K_r2_ interaction) and VDR concentration refined the model enabling fit to experimental data (Figure S1 dotted lines; black line and dotted line are the same in panel B but not in panel A). Subsequently, the 25OHD dose data were assessed. A change in CYP27B1 concentration resulted in a fit with experimental data (Figure S1 black lines). This fit could also be accomplished by raising the CYP27B1 enzyme activity rate (K_cat_) alone or by combinations of increases in CYP27B1 concentration and activity rate.

### Use of the iSS mathematical model to assess the relative importance of intracrine versus endocrine action of vitamin D *in vivo*

1,25(OH)_2_D has the potential to act in both an endocrine and intracrine manner. Circulating levels of 1,25(OH)_2_D generated via the kidneys appear to play a key role in the classical calciotropic actions of the endocrine vitamin D system [2]. By contrast, intracrine, cell-specific conversion of 25OHD to 1,25(OH)_2_D appears to be the most likely mechanism for non-classical actions of vitamin D on cells such as monocytes-macrophages [26]. To investigate the validity of this latter assumption, the iSS model was used to assess the relative impact of 25OHD or 1,25(OH)_2_D as inducers of monocyte CAMP *in vivo* (Table 6). An exclusive intracrine mechanism was assessed by eliminating serum 1,25(OH)_2_D and clamping serum 25OHD at a sufficiency level of 50 nM. An exclusive endocrine mechanism was assessed by eliminating serum 25OHD and clamping serum 1,25(OH)_2_D at a level of 100 pM. In a basal, unstimulated state, both intracrine and endocrine mechanisms predict minimal induction of monocyte-macrophage CAMP. Likewise, in an ‘activation’ setting with elevated VDR and CYP27B1 endocrine induction of CAMP by 1,25(OH)_2_D was also predicted to be minimal. By contrast, under conditions of VDR/CYP27B1 activation, the iSS model predicted intracrine induction of CAMP by 25OHD, with this response being most prominent with low affinity forms of DBP.

**Table 6:** Predicted induction of monocyte expression of CAMP under endocrine (1,25(OH)_2_D only) or intracrine (25OHD only) conditions with varying DBP genotype and levels of activation.

	Both mechanisms		Intracrine mechanism		Endocrine mechanism	
	50 nM 25OHD		50 nM 25OHD		0 nM 25OHD	
Subject	0.1 nM 1,25(OH)_2_D		0 nM 1,25(OH)_2_D		0.1 nM 1,25(OH)_2_D	
Genotype	Basal	Activated	Basal	Activated	Basal	Activated
GC1F/1F	0.010	0.011	0.010	0.010	0.010	0.010
GC1F/1S	0.010	0.012	0.010	0.011	0.010	0.010
GC1F/2	0.010	0.015	0.010	0.012	0.010	0.010
GC1S/1S	0.010	0.019	0.010	0.013	0.010	0.010
GC1S/2	0.010	0.032	0.010	0.019	0.010	0.010
GC2/2	0.010	0.072	0.010	0.036	0.010	0.010

## Discussion

Current guidelines for vitamin D-sufficiency published by the Institute of Medicine are based exclusively on serum levels of 25OHD (50 nM) required for adequate bone health [8]. It is unclear whether this target level will also be relevant to non-classical responses to vitamin D, and findings from association studies have led many researchers to propose a higher level of serum 25OHD (75 nM) for vitamin D sufficiency [1], [7]. In data presented here we show that another important consideration is the amount of 25OHD and/or 1,25(OH)_2_D that is actually available for use within target tissues – in other words the amount of these metabolites that is free from DBP binding.

The potential importance of free 25OHD as a determinant of vitamin D function is illustrated by a recent study of the relationship between serum 25OHD and skeletal health. In this report the authors demonstrated association between bone mineral density (BMD) and levels of free 25OHD but not total serum 25OHD [14]. The implication from these human data was that levels of free 25OHD are a more meaningful marker of the biological impact of vitamin D than total 25OHD. In this study the authors also described association between BMD and ‘bioavailable’ 25OHD (free 25OHD combined with albumin bound 25OHD). The distinction between these two parameters is interesting but no significant difference was noted for associations between free or ‘bioavailable’ 25OHD and BMD [14], possibly because DBP is a relatively abundant steroid hormone binding protein. Because of this, we did not conduct analysis using their definition of ‘bioavailable’ vitamin D in the model we present in this report. However, it is possible that bioavailability versus free steroid may be informative for other steroid-binding globulins such as sex-hormone binding globulin, which is approximately 100-fold less abundant than DBP [27].

At present there is no available technology for rapid and reproducible measurement of free vitamin D metabolites in serum samples. Rather the studies linking free 25OHD with BMD for example [14], relied on estimation of the level of free 25OHD based on existing mathematical models. The eSS model presented here was derived using the same equations employed to estimate free vitamin D in the original studies of this concept [9], [10], as well as the recent BMD association data [14]. Values for free 25OHD and 1,25(OH)_2_D generated by the new eSS models are consistent with previous reports [9], [10]. However, importantly, the new eSS math model we report here incorporates not only DBP serum concentration but also genotypic variations in DBP affinity. The GC1S and GC2 alleles of the DBP gene are derived from the ancestral GC1F allele following two amino acid changes: a D432E change to form GC1S and a T436K change to form GC2. These amino acid changes correlate with decreased affinity of DBP for vitamin D metabolites [13]. The elevated levels of free 25OHD calculated for low affinity forms of DBP such as GC2/2 and GC1S/2 therefore provide an explanation for the relative potency of these forms of DBP in promoting antibacterial responses to 25OHD *in vitro* [19].

The eSS model for estimating free 25OHD is easily implemented with the input of several variables (DBP genotype, DBP concentration, albumin concentration, 25OHD and 1,25(OH)_2_D levels). Since not all of these variables may be available for every subject studied, the model can be used to generate a spectrum of results. For example, in the case of absent DBP genotype, it is possible to generate a range of free 25OHD values from lowest to highest affinity forms of DBP. In the absence of data for serum concentrations of 1,25(OH)_2_D (a non-routine assay), an optimized input value (e.g. 100 pM 1,25(OH)_2_D) can be used instead. However, analysis of the effects of variable levels of 1,25(OH)_2_D suggest that this has very little impact on free 25OHD relative to changes in DBP concentration or binding affinity (data not shown). With these caveats in mind, the eSS model provides an exciting new approach to assessing the true biological vitamin D status of any given individual. Patients with low serum levels of 25OHD may nevertheless exhibit adequate or optimal levels of free 25OHD if they have inherited a low affinity form of DBP. GC allelic combinations such as Gc2/2 not only encode lower affinity binding to 25OHD but also appear to circulate at lower concentrations. In this setting, relatively low levels of total serum 25OHD may support relatively high levels of free 25OHD. By contrast, a high affinity form of DBP would produce relatively low free 25OHD. The latter may be important in ethnic groups such as Africans and African-Americans known to exhibit a higher prevalence of high affinity GC1F/1F DBP [11], where serum levels of 25OHD are commonly low due to darker skin pigmentation and impaired UV-light-induced epidermal synthesis of vitamin D. Under these conditions the eSS model would predict extremely low levels of free 25OHD. Thus, the eSS model may help to evaluate the efficacy of vitamin D repletion strategies by examining both free 25OHD and total 25OHD. Consequently, optimization of vitamin D status in patients with high affinity DBP or high DBP concentrations may require higher levels of supplemental vitamin D than patients with low affinity/low concentration DBP.

The iSS model was developed to further clarify the functional biological impact of DBP-derived variations in free 25OHD. Data in Table 5 show clearly that in the absence of any immune stimulus, there is likely to be very little cathelicidin expression in vivo, irrespective of vitamin D status or DBP genotype. *In vivo*, vitamin D-mediated induction of cathelicidin is only observed when immune stimulation is assumed to result in a 5-fold induction of VDR and 10-fold induction of CYP27B1. However, this effect is much more pronounced for low affinity forms of DBP underlining the importance of DBP as an important factor in defining the efficacy of vitamin D-induced antibacterial activity both in vitro and in vivo. The current model does not take into consideration activity of the vitamin D catabolic enzyme 24-hydroxylase (CYP24A1), which may attenuate the activity of 25OHD and 1,25(OH)_2_D by catabolizing these metabolites [28], [29]. Our model was based on *in vitro* data after 6 hours (Figure 3) where the level of protein expression and enzyme catalytic activity for CYP24A1 is likely to be limited. Another consideration is that the presence of splice variant forms of CYP24A1 [30] that result in catalytically inactive protein may further diminish the impact of 24-hydroxylase activity during the brief duration of immune activated intracrine-driven CAMP expression.

The eSS and iSS mathematical models described in this paper suggest a potential revision of parameters used to define adequate and inadequate vitamin D status. However, the models were also designed to help shed light on the basic mechanisms for vitamin D action. In particular, the affinity of DBP for inactive 25OHD relative to active 1,25(OH)_2_D was used to address a key unresolved question concerning effects of vitamin D metabolites, such as the induction of innate antibacterial activity. Namely, is intracrine metabolism of 25OHD the most effective way for vitamin D to enhance innate immunity or can endocrine levels of 1,25(OH)_2_D achieve the same action? Data shown in Table 6 suggest that intracrine metabolism is a far more effective way of inducing monocyte-macrophage CAMP expression relative to the actions of systemic 1,25(OH)_2_D. Clearly these are data designed for a particular cell type and one specific response to vitamin D. However, given that we have seen similar *in vitro* induction of CAMP in other cell types [17], it is tempting to conclude that similar intracrine pathways will be the optimal mechanism for vitamin D responses at many extra-skeletal sites. This is important given that serum 25OHD levels reflect changes in vitamin D status and may, in turn, affect vitamin D-directed biological activity in many peripheral tissues independent of 1,25(OH)_2_D.

Any attempt at mathematical modeling is constrained by assumptions and these models assume the so-called ‘free hormone hypothesis’ that has been proposed as a general mechanism for the cellular uptake of steroid-like molecules because they are highly lipophilic and therefore have the potential to passively diffuse across cell membranes [21], [31]. However, it is important to recognize that in some circumstances vitamin D and its metabolites utilize other mechanisms. For instance, in renal proximal tubule cells, uptake of 25OHD and subsequent conversion to 1,25(OH)_2_D involves endocytosis of DBP via the megalin and cubilin receptors [15], [32]. This process is fundamental to the generation of circulating, endocrine levels of 1,25(OH)_2_D and provides an explanation for the recent genome-wide association studies of a white European cohort which showed that lower affinity forms of DBP are associated with lower circulating levels of 25OHD [33], [34]. The conclusion from these data is that 25OHD bound to lower affinity forms of DBP is less readily reabsorbed into the proximal tubules and is thus excreted more easily. Additionally, a similar DBP-megalin-mediated endocytosis of 25OHD has also been described for breast epithelial cells [35], [36]. Thus, the ‘free hormone hypothesis’ we incorporated into the eSS model is not necessarily universal.

Although data in this study are focused on non-classical actions of 25OHD, these models could also be applied to cell types engaged in classical actions. Preliminary in vitro data (Figure S1) for 25OHD and 1,25(OH)_2_D mediated induction of osteocalcin mRNA expression in MG-63 osteoblastic bone cells were compared with mathematically predicted values. Data suggests that the model used for adherent monocytes can be utilized in a different cellular context although this requires modification of some VDR and CYP27B1 dependent parameters. Thus, the model may be useful in studying vitamin D action in a variety of settings. For example, the action of synthetic vitamin D analogs could be modeled where it has been shown that their biological activities could be influenced by their differing affinities to DBP and VDR [37]. Finally, we anticipate that the iSS model will be useful in comparing predicted data with experimental observations from wild type and DBP knockout mice. It is not possible to apply our current cathelicidin readout to mice because vitamin D-mediate regulation of this gene is observed only in primates [38]. One study using the DBP knockout mouse assessed macrophage and neutrophil recruitment to a site thioglycolate injection showed that they were normal for this immune function despite their very low serum levels of 25OHD [39]. How these mice respond to a pathogenic challenge would be an important area for future studies.

## Materials and Methods

### Cell culture

Ficoll isolated peripheral blood mononuclear cells (PBMCs) derived from anonymous healthy donors that were screened according to standard blood transfusion protocols were obtained from the Center for AIDS Research Virology Core/BSL3 Facility (supported by the National Institutes of Health award AI-28697 and by the UCLA AIDS Institute and the UCLA Council of Bioscience Resources). Briefly, monocytes were enriched by adherence by incubating 2.5×10^6^ PBMCs per well in 24-well plates for 2 hours in Macrophage Serum Free Media (M-SFM, Invitrogen, Carlsbad, CA). Non-adherent cells were removed by washing with serum-free (SF) RPMI and remaining cells then cultured overnight in RPMI with 10% human AB serum (Omega, Tarzana, CA) supplemented with GM-CSF (10 U/ml; graciously provided by Dr. Modlin. UCLA, Los Angeles, CA). After overnight incubation, cells were washed with SF RPMI and then incubated with RPMI+5% human AB serum (Omega, Tarzana, CA) with varying amounts of 25OHD_3_ (10–300 nM), 1,25(OH)_2_D_3_ (0.04–12 nM) (Biomol, Plymouth Meeting, PA) or with vehicle (0.2% ethanol) for 6 hrs. Figure 3 represented results obtained from triplicate qPCR reactions performed on RNA isolated from each well of dose response culture of adherent monocytes from one donor. The general shape of the curve was typical of dose responses but specific values to guide mathematical model fitting was based on this specific assay run.

### RNA Isolation and Quantitative Real-Time PCR

RNA was isolated by Trizol (Invitrogen, Carlsbad, CA) extraction and cDNA was synthesized by Super Script Reverse Transcriptase III (Invitrogen, Carlsbad, CA) according to manufacturer protocol utilizing random primers. Q-PCR analysis was performed on a Stratagene MX-3005P instrument utilizing TaqMan system reagents from Applied Biosystems (Foster City, CA). Specifically, we utilized FAM-labeled TaqMan Gene Expression Assay probe/primer Hs00189038_m1 (CAMP) in conjunction with VIC/MGB Probe/Primer Eukaryotic 18S rRNA Endogenous Control (part number 4319413E) as the internal calibrator. All cDNAs were amplified under the following conditions: 50°C for 2 min; 95°C for 10 min followed by 45 cycles of 95°C for 15 sec and 60°C for 1 min. Results were reported as ΔΔ Ct values (ΔCt value for vehicle-treated control – ΔCt for treated sample).

### Mathematical Modeling

#### Extracellular steady state (eSS) free-ligand modeling

The free/bound ligand-binding protein model shown in Figure 1 (black reactions) was described previously with mathematical equations for an arbitrary number of ligands and binding proteins [22] and specifically for 25OHD and 1,25(OH)_2_D binding to DBP and Albumin [9], [10], [23]. In general the buffering reactions follow
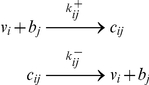
where *v_i_* are ligands, *b_j_* are binding proteins, and *c_ij_* are the complexes of ligands and binding proteins which form at the rate *k^+^_ij_* and dissociate at the rate *k^−^_ij_*. For our system in the extracellular space *v_i_ ∈ {25OHD, 1,25(OH)_2_D}* are the free vitamin D metabolites, and the b*_j_ ∈ {DBP_k_, DBP_l_, Albumin}* are the binding proteins where we solve the system anew for each homo/heterogeneous genotype pair (*k,l*), *k,l ∈ {GC1F, GC1S, GC2}*. In steady state, free vitamin D levels are described by the system of coupled non-linear algebraic equations for each vitamin D metabolite
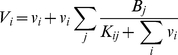
(M1)where *V_i_* and *B_j_* represent total *v_i_* and *b_i_*, respectively, in free and bound forms. *K_ij_* = *k^−^_ij_*/*k^+^_ij_* is the dissociation constant for *v_i_* binding to *b_j_*. The different affinities [13] and expression levels of DBP genotypes [12] and other biochemical parameters [9], [10] are described in Table 1. The equations in (M1) constitute the eSS model. The results of the eSS model for increasing levels of 25OHD or 1,25(OH)_2_D are shown in Figure 2.

#### Intracellular steady state (iSS) model

The intracellular reactions shown in Figure 1 (gray reactions) describe the incorporation of the actions of CYP27B1 and VDR in the cell acting upon and responding to the ligands made available by DBP and Albumin in the blood or general extracellular environment in Figure 1 (black reactions) and calculated by the eSS model. Because the blood volume *in vivo* or extracellular volume *in vitro* is much larger than the intracellular volume (1×10^6^ fold for monocytes in blood), we assume the extracellular levels of free vitamin D, *v^o^*, are little affected by intracellular dynamics. However, *v^o^* acts as a source for the intracellular levels of free vitamin D, *v^c^*. Consequently, in addition to the extracellular free levels (endocrine) we can add the intracellular levels created by balancing free diffusion of vitamin D across the membrane and the dynamics of CYP27B1 action (intracrine). The intracellular component for 25OHD, *v^c^_1_*, then follows from solving for the unique solution of the nonlinear algebraic equation
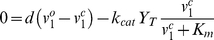
(M2a)where *v^ o^_1_* has been fixed from solving (M1) and we assume the Michaelis-Menten form for enzyme kinetics with unitary Hill coefficient and Michaelis-Menten constant, *K_m_*. The permeability of a monocyte to either 25OHD or 1,25(OH)_2_D is given by the constant *d* and the maximal rate of enzymatic conversion is given by the total amount of CYP27B1, *Y_T_*, times the catalytic rate, *k_cat_*. From the solution to equation (M2a), we calculate the free level of intracellular 1,25(OH)_2_D as
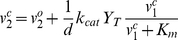
(M2b)where *v^ o^_2_* has also been fixed from solving (M1). The equations (M2a) and (M2b) along with the output of CAMP (described below) constitutes the iSS model. Variables are shown in Table 3 and parameters in Table 4 respectively.

#### Modeling CAMP transactivation

We treat the final production of CAMP as a competitive process between VDR bound 1,25(OH)_2_D and VDR bound 25OHD binding to CAMP-VDRE-DNA (proximal promoter) resulting in CAMP-mRNA transcripts and CAMP itself. We normalize maximal production to 1 plus basal levels (i.e. *η* = 1) with the level of CAMP is given by
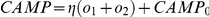
where *o_1_* and *o_2_* are the fractions of active transcription complexes containing 25OHD-VDR and 1,25(OH)_2_D-VDR, respectively, *η* is the net rate of transcription and translation of CAMP relative to the rate of CAMP-mRNA and CAMP degradation, and *CAMP_0_* is the basal CAMP level. The VDR bound to 25OHD, *r_1_*, and VDR bound to1,25(OH)_2_D, *r_2_*, are given as functions of free 25OHD, *v^c^_1_*, and free 1,25(OH)_2_D, *v^c^_2_*, while the active fractions of the CAMP-VDRE-DNA, *o_1_* and *o_2_* are given as functions of *r_1_* and *r_2_*.
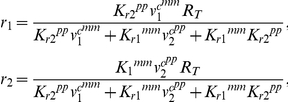


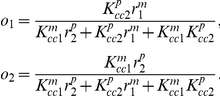



### VDR related parameters

The number of VDR molecules per cell was set at 3000 [40]; thus, basal VDR concentration was calculated to be 1.2 nM assuming monocytes were spherical with a diameter of 20 µm [41]. VDR bound 1,25(OH)_2_D with affinity constant 1/*K_r2_* = 1×10^10^ M^−1^ [42] and we estimated 25OHD bound VDR with affinity constant 1/*K_r1_* = 2×10^7^ M^−1^ which is 500-fold weaker [43]. For this modeling effort, a dissociation constant *K_cc2_* = 1×10^−10^ M was used based on the reported value from VDR/1,25(OH)_2_D binding to VDRE oligonucleotide probes in electrophoretic mobility shift assays [44]. Affinity for actual VDRE targets in chromatin might differ. We assumed that VDR/25OHD was 10-fold less able to bind VDRE (*K_cc1_* = 1×10^−9^ M). Additionally, once VDR/25OHD/VDRE complexes were formed, we assumed they were equally as effective in yielding complete transcripts compared to VDR/1,25(OH)_2_D/VDRE complexes. Thus, the key determinant of sensitivity is the much lower affinity of VDR for 25OHD compared to 1,25(OH)_2_D.

We assume the potential for cooperativity of free 25OHD or 1,25(OH)_2_D in binding to VDR and in VDR/25OHD or VDR/1,25(OH)_2_D in binding to VDRE. We find that a power of 2 (*pp* = 2) in 1,25(OH)_2_D binding to VDR and a power of 2 (*p* = 2) in VDR/1,25(OH)_2_D binding to VDRE to be optimal cooperativity constraining other parameters. We also find that while cooperativity of 25OHD binding to VDR does not seem important (*mm* = 1), there appears to be useful cooperativity of VDR/25OHD binding to VDRE (*m* = 2). We do note that while *m* = 2 works well for both the monocyte and MG-63 cell data, for MG-63 cells *m* = 1 has a better fit at low 25OHD while keeping the model within standard deviation bounds at higher 25OHD levels (data not shown). While *o_1_* does not impact CAMP production in the present study, we retain the term in the model for potential interest in 25OHD rescue of vitamin D dependent activation in the context of CYP27B1 knock-out mice [45].

### CYP27B1 related parameters

The rate of CYP27B1 activity in mitochondria of living cells is unknown but CYP27B1 enzymatic activity has been measured in reconstitution studies with artificial vesicles [46]; thus, we have assumed an enzyme rate of 0.1 µM/hr consistent with that report. The amount of CYP27B1 in cells is also not known. However, based on mathematical fitting of our *in vitro* experimental data and an assumed CYP27B1 rate of 0.1 µM/hr, we estimated the basal amount of CYP27B1 to be 0.1 nM. K_m_ of CYP27B1 was set at 1 µM based on reports measuring the K_m_ between 0.38–2.7 µM [29], [47], [48], [49]. We also assumed the potential for cooperativity in enzymatic conversion of 25OHD to 1,25(OH)_2_D but cooperativity in 25OHD binding to CYP27B1 then also requires 25OHD/VDR affinity to VDRE to be greater than that of 1,25(OH)_2_D/VDR to compensate and fit the in vitro data. This would imply that 25OHD was driving CAMP production rather than 1,25(OH)_2_D, and so coupled with the lack of evidence for CYP27B1 using cooperativity we reject enzyme cooperativity in this case.

### Computational Solution

The eSS and iSS models were solved using Matlab (Mathworks, 2009) and are available upon request of the authors.

## Supporting Information

Figure S1 **Comparison of iSS-predicted effects of 25OHD or 1,25(OH)_2_D on MG-63 osteoblast expression of osteocalcin with observed in vitro dose responses.** MG-63 were incubated for 6 hrs in media containing 2% serum with doses of (A) 25OHD (0–200 nM) and (B) 1,25(OH)_2_D (0–20 nM) and osteocalcin expression (ΔΔCt) was determined by qPCR. In each case, experimental data are indicated by blue dots and error bars (± SD) and reflect two biological treatment replicates and three qPCR determination replicates of each biological sample. Dashed lines indicate data produced by the iSS mathematical model using monocyte parameters. Dotted lines indicate model after adjustment of parameters to permit fitting to 1,25(OH)2D experimental data. Black lines indicate model after adjustment of parameters to permit fitting to 25OHD experimental data. Please note that dashed and black lines are the same in (B) but not (A). For the purpose of this modeling, DBP was represented by the GC1F/1F allelic combination.
(TIF)

Text S1 **Material and Methods for Figure S1.**
(DOC)
