# Supplementary figures and images for: Vitamin D Binding Protein and Monocyte Response to 25-Hydroxyvitamin D and 1,25-Dihydroxyvitamin D: Analysis by Mathematical Modeling
Source: PLoS One. 2012 Jan 24;7(1):e30773. doi: 10.1371/journal.pone.0030773 (PMC3265504; doi:10.1371/journal.pone.0030773)

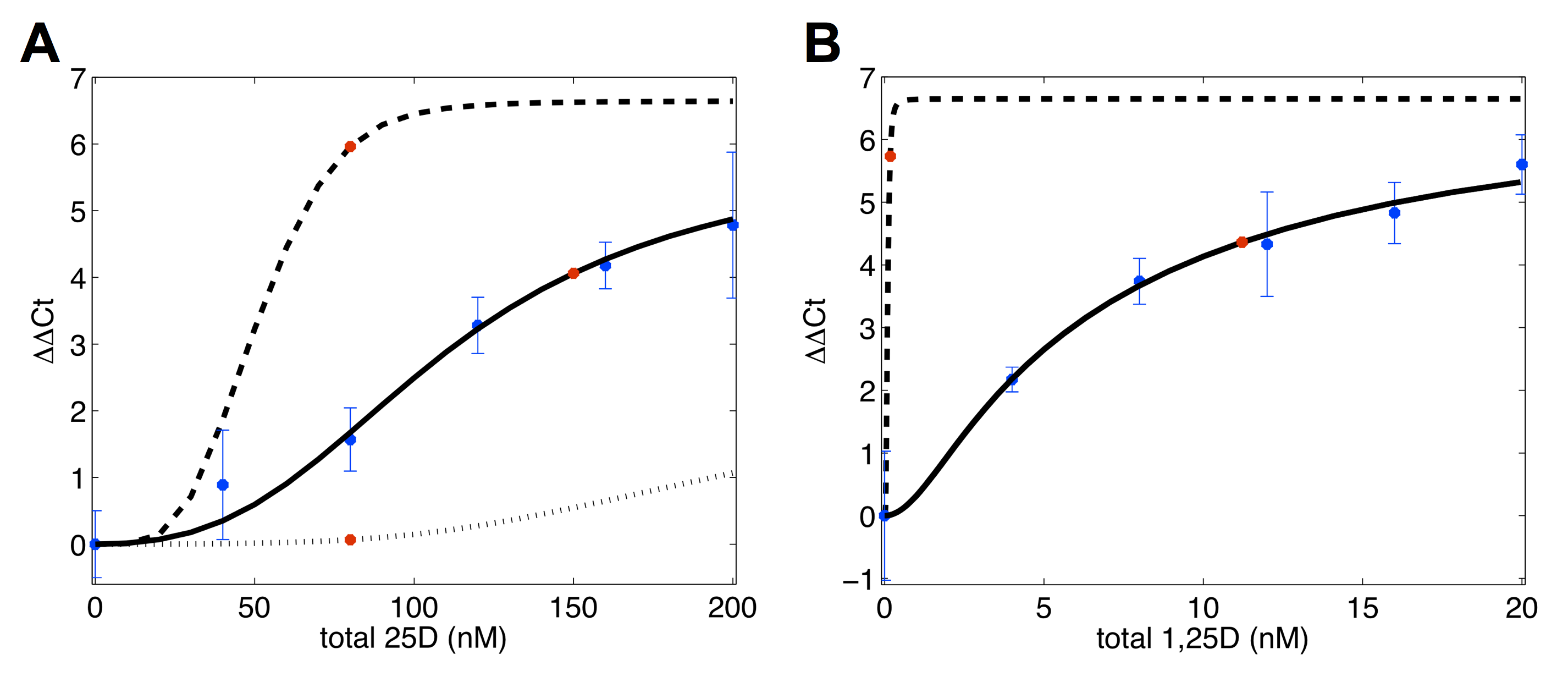

Supplement: Figure S1 — Comparison of iSS-predicted effects of 25OHD or 1,25(OH)2D on MG-63 osteoblast expression of osteocalcin with observed in vitro dose responses. MG-63 were incubated for 6 hrs in media containing 2% serum with doses of (A) 25OHD (0–200 nM) and (B) 1,25(OH)2D (0–20 nM) and osteocalcin expression (ΔΔCt) was determined by qPCR. In each case, experimental data are indicated by blue dots and error bars (± SD) and reflect two biological treatment replicates and three qPCR determination replicates of each biological sample. Dashed lines indicate data produced by the iSS mathematical model using monocyte parameters. Dotted lines indicate model after adjustment of parameters to permit fitting to 1,25(OH)2D experimental data. Black lines indicate model after adjustment of parameters to permit fitting to 25OHD experimental data. Please note that dashed and black lines are the same in (B) but not (A). For the purpose of this modeling, DBP was represented by the GC1F/1F allelic combination. (TIF) [file pone.0030773.s001.tif]
