# Supplementary material for: Vitamin D Binding Protein and Monocyte Response to 25-Hydroxyvitamin D and 1,25-Dihydroxyvitamin D: Analysis by Mathematical Modeling
Source: PLoS One. 2012 Jan 24;7(1):e30773. doi: 10.1371/journal.pone.0030773 (PMC3265504; doi:10.1371/journal.pone.0030773)
Supplement: Text S1 — Material and Methods for Figure S1. (DOC) [file pone.0030773.s002.doc]

# Supporting Information

**Material and Methods for Figure S1.**

*Cell culture*

50,000 MG-63 cells per well were seeded into 12-well plates. Two days later, doses of 25OHD_3_ (0 to 200 nM) or 1,25(OH)_2_D_3_ (0 to 20 nM) were added for 6 hour incubation in 2% human serum supplemented DMEM.

*RNA Isolation and Quantitative Real-Time PCR*

RNA was isolated, cDNA prepared and Q-PCR analysis performed as described in main Material and Methods section with the exception of utilizing FAM-labeled TaqMan Gene Expression Assay probe/primer Hs01587814_g1 to determine osteocalcin/BGLAP gene expression.

*Mathematical Modeling*

Math modeling was performed as described in the main Material and Methods section with adjustment of DBP and albumin levels to correspond to 2% serum. Math model fitting to in vitro results was performed. Considering 1,25(OH)_2_D_3_ dose response experimental data resulted in primary adjustments to K_r2_, K_cc2_, p and VDR concentration parameters. Addressing the 25OHD_3_ dose response experimental data resulted in a secondary adjustment to CYP27B1 concentration parameter. Adjustment of CYP27B1 K_cat_ alone or a combination of adjustments to activity rate and concentration could achieve the same fit.

| Parameter | Adherent monocyte modeling | MG-63 modeling |
| --- | --- | --- |
| K_r2_ | 1 x 10^-4^ | 1 x 10^-2^ |
| K_cc2_ | 1 x 10^-4^ | 1.7 x 10^-4^ |
| p | 2 | 1 |
| VDR concentration | 1.2 x 10^-3^ | 4.94 x 10^-4^ |
| CYP27B1 concentration | 1 x 10^-4^ | 2.17 x 10^-3^ |
